# Financial Performance Analysis in European Football Clubs

**DOI:** 10.3390/e22091056

**Published:** 2020-09-21

**Authors:** David Alaminos, Ignacio Esteban, Manuel A. Fernández-Gámez

**Affiliations:** 1 Department of Financial Management, Calle de Alberto Aguilera 23, Universidad Pontificia Comillas, 28015 Madrid, Spain; 2 PhD Program in Economics and Business, Campus de El Ejido, s/n, University of Málaga, 29071 Málaga, Spain; iesteban@uma.es; 3 Department of Finance and Accounting, Campus de El Ejido, s/n, University of Málaga, 29071 Málaga, Spain; mangel@uma.es

**Keywords:** financial performance, football clubs, neural networks, multilayer perceptron, quantum neural network

## Abstract

The financial performance of football clubs has become an essential element to ensure the solvency and viability of the club over time. For this, both the theory and the practical and regulatory evidence show the need to study financial factors, as well as sports and corporate factors to analyze the possible flow of income and for good management of the club’s accounts, respectively. Through these factors, the present study analyzes the financial performance of European football clubs using neural networks as a methodology, where the popular multilayer perceptron and the novel quantum neural network are applied. The results show the financial performance of the club is determined by liquidity, leverage, and sporting performance. Additionally, the quantum network as the most accurate variant. These conclusions can be useful for football clubs and interest groups, as well as for regulatory bodies that try to make the best recommendations and conditions for the football industry.

## 1. Introduction

Financial performance is the main concern that affects any company, where the importance of control will increase in the new post-COVID scenario, which will cause major financial performance problems for companies after large drops in consumption. Typically, these financial performance analyses and models have been done in a limited way, focusing on certain sectors and types of companies. This has evidenced the lack of effective and specific models that control the level of financial performance and its analysis, like in the football industry [1].

Recently, a considerable number of European football clubs showed a difficult financial situation after suffering declines in their financial performance, declaring losses due to poor performance. Recently, the Union of European Football Associations (UEFA) investigated roughly 80 clubs for this matter [2]. Sports competitiveness can be seriously affected by the losses suffered by the club, which could make it impossible to attract talent in players, as well as make it difficult to plan new investments in infrastructure [3,4]. Therefore, previous literature has pointed out that the balance of the club’s profit and loss account is the variable that best explains the financial position of European club football [5,6]. To mitigate these potential future financial problems for clubs, UEFA introduced the financial fair play (FFP) financial regulation as one more element of its licensing regulations [7]. This concept aims to reduce the default of debts to other clubs or employees and financial doping with non-organic financing, putting the focus on the control of the financial statements of the clubs. This means that football clubs must generate their income organically and not receive money from agents outside the football activity to remain as competitive clubs, as this does not reflect a real financial performance situation.

Different authors have analyzed the financial performance of football clubs, although carrying out broad objectives and in the same way with the type of variables used to analyze financial performance and income generation [8]. According to [9], it is the usual to obtain more reliable and more personalized samples if only a single business sector is focused on, so more robust models can be created. Existing models on the financial performance analysis have been designed from a reduced number of financial variables, initially ruling out other combinations of indicators that may be decisive for studying financial performance. Besides, the literature asks that non-financial variables be added that show the behavior of the market where the company operates and, therefore, its possible sources of income [9,10,11,12]. However, with the FFP in 2011, concerns about the financial situation of the clubs are focused on the losses generated, and in the generation of income organically through the activity of football and related activities. However, there are no models designed to analyze the behavior of the financial performance of football clubs, and the literature demands this kind of model for analyzing financial performance to adjust to the financial, marketing, and legal requirements raised in these years [12,13,14].

This work creates a new financial performance analysis model for football clubs to respond to the growing needs regarding the financial situation in the management of football clubs. A sample of European football clubs was used, which includes two measures of financial performance for clubs, such as return on net worth (RONW) and return on capital employed (ROCE). This dataset collects both the sporting performance situation and the financial situation for the years 2016–2018 and has been obtained using multilayer perceptron and quantum neural networks as a methodology. Neural network techniques are highly popular in financial performance analysis due to their high robustness [10]. Due to this methodology, the results showed conclusions that can be useful for soccer administrative bodies and any other interest group in football clubs.

Therefore, this work improves on the previous literature in the improvement and expansion of explanatory variables to forecast the financial performance of football clubs, considering new types of variables not yet contrasted previously and that gave good results. This has great implications for football managers and executives, as they will be able to use this information to perform a more accurate analysis of financial performance. Along the same lines, we improved the precision of the analysis compared to what was obtained in the literature after the use of our innovative methodology.

The present study is organized as follows: Section 2 develops a review of the literature on financial performance in football clubs. In Section 3 explain the methodology applied. Section 4 explains the data and the variables used in the research and Section 5 the results obtained are analyzed. Lastly, the conclusions and their implications are explained.

## 2. Literature Review

Rohde and Breuer [3] constructed an empirical model of financial performance to apply it to a data set of the top 30 European football clubs in revenue during the period 2004–2013. The conclusions expose that national and international sporting success assure the financial success, as well as for the value of the brand; in turn, sporting success is driven by investments in players, and the majority of investments in players tend to be driven by private and foreign investors. López-Busto et al. [9] studied the relevant management variables that explain the economic results of Spanish football clubs in the first division. They performed a regression analysis with panel data to find out how the proposed models were verified. The work concludes that individually the benefits obtained in the previous season, the number of games played in European competitions, and the position reached in the league classification significantly affect the net result. Likewise, the number of European players, the financial expenses, and the net result of the previous season act as a joint model that would affect the profit possibilities of the clubs.

Carmichael et al. [14] investigated the relationship between sporting success and commercial success in European football. They used data from Premier League clubs, applying both financial variables and skill indicators. They concluded that investment in highly skilled players, with increasingly wealthy teams capable of sustaining or even building on success by spending more on players than less successful clubs, maintained a high level of financial performance. To the extent that the richest clubs succeed in their goal, there is a causal link between the income earned and the competitive imbalance through investments in players. Giovanni [10] presented an optimization model with the objective of the expected benefits. The model ensures that the team has the required a skill mix with other legal requirements such as budget limits. Using data from Premier League clubs, their results showed that reported team value growth is driven by investments in young players.

Andreff [13] analyzed the connection between the financial and sporting performance of French football clubs. He examined the financing sources of professional football and the role of the league’s auditing body. They also analyzed some significant elements of financial performance, highlighting the soft budget constraint of football clubs, helping to create a circle between the increasing commercial value of TV broadcasting rights and increasing the salaries of football clubs. players. Galariotis et al. [8] proposed a two-stage method to conduct a multicriteria analysis to rank clubs on their financial and business performance dimensions. They found a positive relationship between business performance and sporting performance, and a one-way inverse relationship where financial performance affects sporting performance. In other words, more income positively affects athletic achievements, and these in turn positively impact income. Having a higher level of income does not help financial performance as this can be subjectively analyzed by stakeholders, prioritizing short-term sporting objectives over long-term viability [4]. Added to this is the scarcity of data on player salaries, making the econometric test of the possible connection more complicated. shows that the competition to recruit talent generates excessive demand from great players and makes the club’s financial performance levels negative.

Dimitropoulos and Limperopoulos [15] analyzed the existence of a possible connection between sports and financial performance with data from Greek clubs and how investment in recruiting new players also affects this connection. They used a panel data methodology, obtaining results that concluded that the greater the investment in players, the more successful the club was on the field. However, this increased spending on recruiting players made the club less profitable and insolvent, suggesting that these decisions are not based on economic standards, but rather prioritize sports results. They also influence a greater investigation of the financial ratios to analyze the financial performance of the clubs. da Costa Jahara [12] aimed to develop an index to analyze the financial performance of football clubs in Brazil with data from 2014. They used indicators of liquidity, profitability, and indebtedness, in addition to the analysis of the solvency of the clubs. As a result, they showed that the clubs present low financial performance when analyzed individually both in the analysis of liquidity, indebtedness, profitability, and solvency indicators. However, this result cannot explain the competitive performance of the teams in this championship, showing problems in the study of financial variables. Iconomescu [11] studied the sporting and financial performance of various Romanian clubs for the period 2010–2015. Romanian law classifies football clubs as joint-stock companies or NGOs, with strict rules and regulations for their creation and management. The conclusions of this work showed that a good part of the football clubs obtained large losses, which caused a scenario of low profits and even insolvency. The only football clubs that achieved good sporting and financial performance are those that are privately established and managed. However, those clubs that are constituted as NGOs, financed with donations, are not sustainable in the long term, and face possible economic problems and therefore can cause poor sports performance. Table 1 describes a summary of the differences and advantages of this study with respect to other previous works.

## 3. Neural Networks Methods

### 3.1. Multilayer Perceptron (MLP)

When we talk about neuronal systems, it is important to define the concept of an artificial neuron, a basic element of this type of system, whose description is inherited from the neurobiological principles that describe the behavior of neurons in the cerebral cortex. The artificial neuron consists of input and output elements that are processed in the central unit, as well as the processing elements that will allow the neuron to generalize and learn concepts. From this basic structure, the neuron can map the inputs to obtain at the output, the desired response that could belong to a certain function, and that, due to the activation function that generates it, can fall into two categories. This response depends firstly on the inputs of the neuron and secondly on the operations carried out within the neuron [16,17].

The design of the multilayer perceptron implies the determination of the activation function to be used, the number of neurons, and the number of layers of the network. As mentioned above, the choice of the activation function is usually made based on the desired path, and the fact of choosing one or the other generally does not influence the ability of the network to solve the problem. Regarding the number of neurons and layers, some of these parameters are given by the problem and others must be chosen by the designer. Thus, for example, both the number of neurons in the input layer and the number of neurons in the output layer are given by the variables that define the problem. In some practical applications, there is no question about the number of inputs and outputs. However, there are problems where the number of input variables relevant to the problem is not exactly known. In these cases, many variables are available, some of which may not provide relevant information to the network, and their use may complicate learning since it will involve large architectures with high connectivity. In these situations, it is convenient to carry out a prior analysis of the most relevant input variables to the problem and discard those that do not provide information to the network. This analysis can become a complicated task and require advanced techniques, such as techniques based on correlation analysis, principal component analysis, relative importance analysis, and sensitivity analysis, or techniques based on genetic algorithms, among others. The number of hidden layers and the number of neurons in these layers must be chosen by the designer. No method or rule determines the optimal number of hidden neurons to solve a given problem. In most practical applications, these parameters are determined by trial and error. Starting from an architecture that has already been trained, changes are made by increasing or decreasing the number of hidden neurons and the number of layers until an adequate architecture is achieved for the problem to be solved, which may not be optimal, but which provides a solution.

Let it be a multilayer perceptron with *C* layers—*C* 2 hidden layers—and *n_c_* neurons in layer *c*, for *c* = 1,2, …, *C*. Let *W^c^* = ($w_{ij}^{c}$) be the weight matrix where $w_{ij}^{c}$ represents the weight from the connection of neuron *i* of layer *c* to *c* = 2, …, *C*. We will denote as $a_{i}^{c}$ to the activation of neuron *i* of layer *c*. These activations are calculated as follows:

First, the activation of input layer neurons ($a_{i}^{1}$). The neurons of the input layer are responsible for transmitting the signals received from the outside to the network. So:

(1) $$a_{i}^{1}=x_{i}\;for\;i=1,2,\ldots ,n_{i}$$

where *X* = (*x*_1_; *x*_2_;::::; *x_n_*_1_) represents the vector or pattern of input to the network.

Second, activation of the neurons of the hidden layer *c* ($a_{i}^{c}$): The hidden neurons of the network process the information received by applying the activation function f to the sum of the products of the activations received by their corresponding weights, that is:

(2) $$a_{i}^{c}=\left(\overset{n_{c-1}}{\underset{j=1}{\sum }}w_{ji}^{c-1}a_{ji}^{c-1}+u_{i}^{c}\right)\;\;\;for\;i=1,2,\ldots ,n_{c};\;c=2,3,\ldots .,C-1$$

where *Y* = (*y*_1_, *y*_2_, …, *y_nC_*). The function *f* is the so-called activation function. For this multilayer perceptron, we used the sigmoidal function. These functions have as an image a continuous interval of values within the intervals [0; 1]:

(3) $$f_{sigm}\left(x\right)=\frac{1}{1+e^{-x}}$$

The neural networks used in this project used the supervised learning paradigm and the error correction algorithm, sometimes known as the delta rule. When we speak of supervised learning, we refer to the type of training in which the system is provided with information on the inputs as well as the expected outputs or destinations corresponding to said inputs so that the system has the destinations as a point reference to evaluate its performance based on the difference of these values and modify the free parameters based on this difference.

The equation of the minimization of error is composed of learning patters {(*x*_1_, *y*_1_), (*x*_2_, *y*_2_) *…* (*x_p_*, *y_p_*)} and an error function *ε* (*W*, *X*, *Y*), where the training process tries to seek the weights that minimize the learning error *E* (*W*), as is shown in (4).

(4) Ewmin(W)= wmin ∑i=1pε(W,xi,yi)

Regarding the number of neurons and layers, some of these parameters are given by the problem and others must be chosen by the designer. Thus, for example, both the number of neurons in the input layer and the number of neurons in the output layer are given by the variables that define the problem. In some practical applications, there is no room for doubt about the number of inputs and outputs. However, there are problems where the number of input variables relevant to the problem is not exactly known. In these cases, a large number of variables are available, some of which may not provide relevant information to the network, and their use could complicate learning since it would imply large-scale architectures with high connectivity. In these situations, it is convenient to carry out a preliminary analysis of the input variables most relevant to the problem and discard those that do not provide information to the network. This analysis can become a complicated task and requires advanced techniques, where the main technique is the sensitivity analysis [17]. Furthermore, and for MLP to be able to report on the importance of each variable in the results of the constructed model, it is possible to perform a sensitivity analysis [18]. This sensibility analysis starts from the total data to divide this database into groups, and each group works on the network as many times as there are model variables. As soon as the value of one of the variables changes, a value of zero is placed. This can be done because the network works by evaluating your responses against already known ranking values, after defining the expression (5).

(5) $$S_{x_{i}}=\sum_{j=1}^{n}{\left(\Phi x_{ij}\left(0\right)-\Phi x_{ij}\right)}^{2}$$

where $\;\Phi x_{ij}\left(0\right)$ is the value of the network output when the variable *X_i_* is zero, $\Phi x_{ij}$ is the known classification value, *X_i_* is the significant variable, and *Sx_i_* is the sensitivity result of each variable.

### 3.2. Quantum Neural Networks (QNN)

The QNN is built from quantum computation techniques. Qubit is defined as the smallest unit of information in quantum computation, which is a probabilistic representation. A qubit may either be in the “1” or “0” or any superposition of the two [19]. The state of the qubit can be defined as follows:

(6) |Ψ〉=α|0〉+β|1〉

where α and are the numbers that point out the amplitude of the corresponding states such that ${\left|\alpha \right|}^{2}+{\left|\beta \right|}^{2}=1$. A qubit is defined as the smallest unit of information in quantum computation. It is determined as a pair of numbers $\left[\begin{array}{c} \alpha \\ \beta \end{array}\right]$. An angle *θ* is a specification that represents geometrical aspects and is defined such that: *cos*(*θ*) =|*α*| and *sin*(*θ*) =|*β*|. Quantum gates may be applied for adjusting the probabilities because of weight upgrading [19]. An example of a rotation gate can be:

(7) $$U\left(\Delta \theta \right)=\left[\begin{array}{c} cos\left(\Delta \theta \right)\\ sin\left(\Delta \theta \right) \end{array}\begin{array}{c} -sin\left(\Delta \theta \right)\\ cos\left(\Delta \theta \right) \end{array}\right]$$

A state of the qubit can be upgraded by applying the quantum gate explained previously. The application of the rotation gate on a qubit is defined as follows:

(8) $$\left[\begin{array}{c} \alpha^{\prime }\\ \alpha^{\prime } \end{array}\right]\left[\begin{array}{c} cos\left(\Delta \theta \right)\\ sin\left(\Delta \theta \right) \end{array}\begin{array}{c} -sin\left(\Delta \theta \right)\\ cos\left(\Delta \theta \right) \end{array}\right]\left[\begin{array}{c} \alpha \\ \beta \end{array}\right]$$

The hybrid quantum-inspired neural network is begun with a quantum hidden neuron from the state |0〉, preparing the superposition as:

(9) $$\sqrt{p}|0+\sqrt{1-p}|1\;with\;0\leq \left|p\right|\leq 1$$

where *p* represents the random probability of initializing the system in the state ∣0〉. The desired state can be reached by using rotation gate *R*:

(10) $$R\left(\theta \right)=\left[\begin{array}{c} cos\left(\Delta \theta \right)\\ sin\left(\Delta \theta \right) \end{array}\begin{array}{c} -sin\left(\Delta \theta \right)\\ cos\left(\Delta \theta \right) \end{array}\right]$$

(11) $$tan\left(\theta \right)=\sqrt{\frac{p}{1-p}}$$

(12) $$\theta =arctan=\sqrt{\frac{p}{1-p}}$$

(13) $$R\left(\theta \right)=\left[\begin{array}{c} \sqrt{1-p}\\ \sqrt{p} \end{array}\begin{array}{c} -\sqrt{p}\\ \sqrt{1-p} \end{array}\right]$$

(14) $$\left[\begin{array}{c} \alpha \\ \beta \end{array}\right]=\left[\begin{array}{c} \sqrt{1-p}\\ \sqrt{p} \end{array}\begin{array}{c} -\sqrt{p}\\ \sqrt{1-p} \end{array}\right]\left[\begin{array}{c} 1\\ 0 \end{array}\right]$$

The classical neurons are initiated by random number generation. The output from the quantum neuron is determined as follows:

(15) $$v_{j}=f\left(\overset{n}{\underset{i=1}{\sum }}w_{ij}*x_{i}\right)$$

where *f* is a problem-dependent sigmoid or Gaussian function. The output from the network is represented as:

(16) $$y_{k}=f\left(\overset{l}{\underset{j=1}{\sum }}w_{jk}*v_{j}\right)$$

The desired output is the *o_k_* corresponding squared error, which is:

(17) $$E_{k}^{2}=\frac{1}{2}{\left|y_{k}-o_{k}\right|}^{2}$$

The learning follows the rules of the feedforward backpropagation algorithm. The upgrading of the output layer weight is defined as follows:

(18) $$\Delta w_{jk}=\eta e_{k}f^{\prime }v_{j}$$

The weights are upgraded by a quantum gate as appears in Equation (6), so in this case, the equation would be:

(19) $$\left[\begin{array}{c} \alpha_{ij}^{\prime }\\ \beta_{ij}^{\prime } \end{array}\right]=\left[\begin{array}{c} cos\left(\Delta \theta \right)\\ sin\left(\Delta \theta \right) \end{array}\begin{array}{c} -sin\left(\Delta \theta \right)\\ cos\left(\Delta \theta \right) \end{array}\right]\left[\begin{array}{c} \alpha_{ij}\\ \beta_{ij} \end{array}\right]$$

where $\Delta \theta_{ij}=-\frac{\partial E}{\partial \theta_{ij}}=-\frac{\partial E}{\partial y_{k}}\frac{\partial y_{k}}{\partial v_{j}}\frac{\partial v_{j}}{\partial \theta_{ij}}=-E_{k}f^{\prime }w_{jk}v_{j}x_{i}\left(\cos\left(\gamma_{ij}\right)-\sin\left(\gamma_{ij}\right)\right)$ obtaining this result using the chain rule. The variable $\gamma_{ij}$ is a step of $|\Psi_{ij}\rangle$ such that: $|\Psi_{ij}\rangle =\left[\begin{array}{c} cos\left(\gamma_{ij}\right)\\ sin\left(\gamma_{ij}\right) \end{array}\right]$ to develop the last step of $\gamma_{ij}$ should be $\gamma_{ij}^{\prime }=\gamma_{ij}+\eta \Delta \theta_{ij}$; η is the learning rate [19]. This ratio usually takes the value 0.1.

### 3.3. Sensitivity Analysis

For machine learning techniques it is necessary to quantify the significance of the variables that can complete the information offered by the results. For this, a sensitivity analysis was applied, which attempts to quantify the relative importance of the independent variables concerning the dependent variable [20]. As an important step, this analysis eliminates the least important variables, after considering as more important those that show a variance greater than the total variance of the set of varials used. This type of sensitivity analysis is also known by the Sobol method [20] where the variance of total *V* (*Y*) obtained by the equations defined in (20) is decomposed.

(20) $$V\left(Y\right)=\underset{i}{\sum }V_{i}+\underset{i}{\sum }\underset{j}{\sum }>1V_{ij}+\ldots +V_{1,2,\ldots ,k}$$

where *V_i_* = *V*(*E*(*Y*|*X_i_*) and *V_ij_* = *V*(*E*(*Y*|*X_i_*, *X_j_*)) − *V_i_* − *V_j_*.

*S_i_* = *V_i_*/*V* and *S_ij_* = *V_ij_*/*V* define the sensitivity indexes, *S_ij_* being the effect of interaction between two variables. The Sobol method performs the computing of a sensitivity index *STi*, measuring the sum of the sensitivity effects related to the independent variable.

## 4. Data and Variables

This study employed a sample of 234 European professional football clubs that have participated in the first and second division of the national league in 2016 (Appendix A). As dependent variables to study the financial performance of football clubs, two measures widely used by the literature were chosen [8,10,12]. RONW is represented as profits available to equity shareholders/equity shareholders’ funds of corporate, and ROCE is represented as earnings before interest and tax (EBIT)/capital employed.

The financial and corporate governance data of the clubs in the sample were extracted from the Amadeus database of Bureau Van Dijk, according to the criteria used in the previous literature [10,13,14,15]. The sporting performance information was extracted from the Transfermarkt web portal (see: https://www.transfermarkt.com/) [3]. The corporate reputation information was extracted from the clubs’ official accounts of the social networks [8,9,11]. Table 1 displays the concept of the independent variables.

Additionally, to verify the level of reliability of the built models, different test samples were created, and not related to those used in the estimation of the models with data from 2016. The collected data set was classified into three groups to perform the training step with 70% of the observations, the validation step with 10% of the observations, and the testing step with 20% of the observations. The data dedicated to the training step was used to create the model. The validation step was dedicated to evaluating the existence of excessive training. The last step, the testing process, was dedicated to assessing the precision of the models [21]. The sample data for 2017 (*t* + 1) and 2018 (*t* + 2) were reserved for the later estimates section. Table 2 points out the set of independent variables used in this study.

## 5. Results

### 5.1. Descriptive Statistics

Table 3 shows the descriptive statistics for the dependent and independent variables. The RONW value of the sample stood at 10.4%, showing a standard deviation of 9.2%. For its part, the ROCE stood at 2.3%, showing a standard deviation of 3.7%. The differences in terms of RONW and ROCE implied that the sample was characterized by profitability heterogeneity among the countries. On the other hand, the mean reputational variables sampled was 1,974,872 (standard deviation 55,865), with some heterogeneity between social networks. Finally, the financial variables also show some differences between different factors depending on their nature (liquidity, leverage, assets, etc.), but a relatively low standard deviation.

### 5.2. Estimated Results

**Figure 1:**
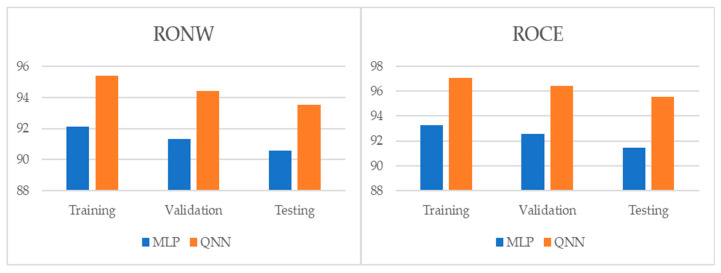
Results of accuracy evaluation: classification (%).

**Figure 2:**
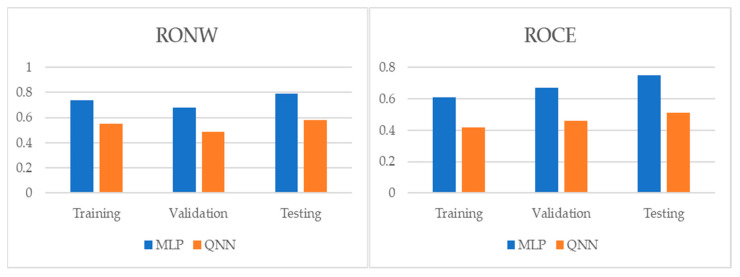
Results of accuracy evaluation: the root mean square error (RMSE).

**Figure 3:**
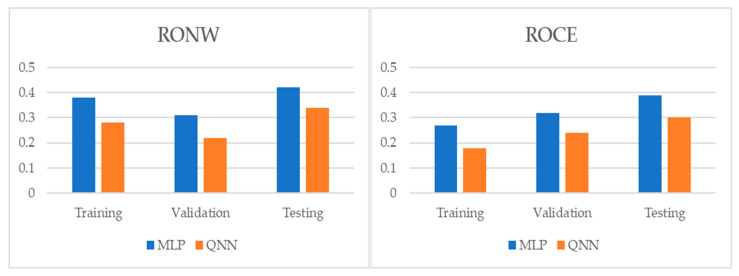
Results of accuracy evaluation: mean absolute percentage error (MAPE).

Table 4 and Table 5, and Figure 1, Figure 2 and Figure 3 show the adjustment level using accuracy, the root mean square error (RMSE), and the mean absolute percentage error (MAPE). In all models, the level of accuracy always exceeded 90.57% for testing data. For its part, the RMSE and MAPE levels were adequate. The model with the highest accuracy was that of quantum neural networks (QNN) with 96.17% for the ROCE variable, followed by the model of deep neural decision trees (DNDT) method with 95.38% for the RONW variable. Taken together, these results provide a level of accuracy far superior to that of previous studies. Thus, in the work of [8], an accuracy of around 77% is revealed, in the work of [8] it is close to 72%, and in the study of [11], it approaches 68%. Finally, Table 6 shows the most significative variables by methods after applying the Sobol method for the sensitivity analysis.

Table 5 shows additional information on the significant variables. Wage Bill (P8) and Current Liabilities/Current Assets (F1) have been significant in the two models for each method used. This demonstrates that the importance shows a higher average expenditure on salaries in football clubs that have a high level of financial performance, different from the previous literature in financial performance [9]. Although other theoretical studies indicate that those who spend more on salaries are teams that compete better to attract talent and improve their sports and marketing results [8,12]. For its part, the variable F1 shows the importance for a club of maintaining a liquidity margin, showing itself as an important variable in previous studies, where some research studies observed the existence of a significant negative relationship between liquidity and financial performance [10,13]. The best results were obtained by the QNN method, where in addition to the variables, CEO (Chief Executive Officer) duality (I4) and the number of followers on Facebook (R1), among others, were also significant for RONW. This shows that the decision-making power of the club’s CEO can be detrimental to the club if it is excessive, while the number of followers on social networks such as Facebook is positively correlated with a higher level of income. These results have been shown by previous theoretical works [12,15], but they had not yet been confirmed in empirical studies on financial performance in the football industry [3,10,15]. For its part, for the ROCE model built with QNN, the variable I4 has also been significant, but there are other important variables such as, for example, EBIT/total assets (F7) and debt coverage ratio (F20). These variables show the importance of profitability on assets of the clubs, while it is also shown that those clubs that do not show difficulties in paying their debts and are concerned about it have a stable level of financial performance. These variables had not yet been shown as significant in previous empirical studies [8,11,14].

On the other hand, the models built by the MLP method show high levels of precision, although lower than those obtained by QNN. Furthermore, these methods show some different significant variables. Such is the case for RONW of the variables of the performance ratio (P7), a variable popularly used to measure the relative sporting performance of a club concerning the rest of the professional clubs in its national league. In this case, it shows that the clubs with the highest sports performance had a higher financial performance. Along the same lines, the variable of the main club in the city (P1) shows the importance of being the most important club in the city, since the club enjoys a greater share of the local market and therefore, a higher level of income. These variables were only significant in studies where other financial concerns of football clubs were studied [21]. For the case of the ROCE model with the MLP method, for the level of the future trend that financial performance could have, although with divergences in previous works regarding its significance to analyze the financial performance, there were some works that show that this variable is not significant [10,13]. Finally, the variable net capital/equity (F14) demonstrated the importance of keeping available and using its capital with which to generate income, which increases the profitability of the club, something already analyzed by other recent works [10,12].

This set of variables observed as being significant represents a group of novel factors that determine the financial performance in football clubs and therefore, different from that shown in the previous literature.

Finally, our methodologies show a precision of a range of 90.57–94.53% of correctness globally for the two dependent variables used. While other works such as [10] achieved an accuracy of 76% in the analysis of the stochastic programming method. For its part, [1] obtained a precision of 73% applying a panel regression. Additionally, using panel methodology, [16] reached an accuracy of 78% for the financial performance analysis with Greek clubs. For the French case provided with the work of [8], after using various methodologies such as the multicriteria analysis and the partial least squares structural equation modeling approach, where they showed a precision of 77% and 87%, respectively. Continuing with the French case, [4] used linear regression to also analyze financial performance in the French league, obtaining an accuracy of 75%. Concerning the Spanish experience, [9] obtained a 71% precision through regression analysis. Finally, [1] used a discriminating analysis with data from Turkey, where they achieved an 81.2% precision capacity.

### 5.3. Post-Estimations

**Figure 4:**
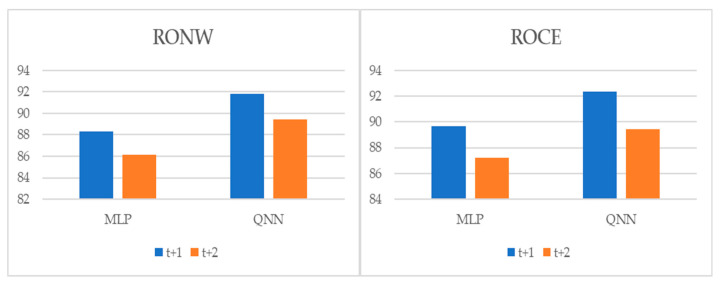
Multiple-step ahead forecasts in the forecast horizon: accuracy.

**Figure 5:**
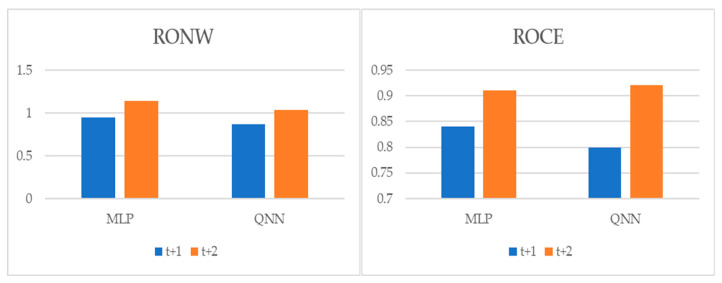
Multiple-step ahead forecasts in the forecast horizon: RMSE.

**Figure 6:**
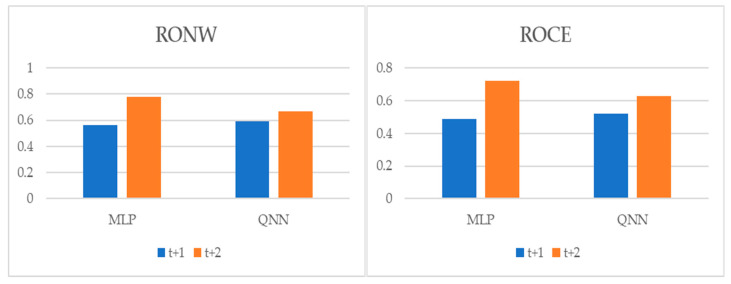
Multiple-step ahead forecasts in the forecast horizon: MAPE.

To perform multiple-step-ahead prediction to obtain a greater robustness of results, we applied an iterative strategy. For this, we trained the models for the prediction of one step and two forward steps, that is, of the moments *t* + 1 and *t* + 2 [22]. These projected data for *t* + 1 and *t* + 2 were included in the data sample as actual observations. Table 7, and Figure 4, Figure 5 and Figure 6 pointed out the accuracy and residual results (RMSE and MAPE) for one-year and two-year forecasting horizons. For *t* + 1, the range of precision for the two techniques was 88.32–91.85% on overall, being in the model of QNN where the percentage of accuracy was higher (91.85%) for the RONW dependent variable. Along the same lines, for the ROCE variable, the precision range was 89.67–92.38%, with QNN being again the methodology with the highest precision (92.38%). For *t* + 2, this range of precision was 86.16–89.41%, being also the method of QNN that the percentage of accuracy was higher (89.41%) for the RONW variable. Additionally, in *t* + 2 for variable ROCE, it again confirmed the predictive superiority of QNN (89.41%) over MLP (87.21%). These results show the high precision and great robustness of the models.

## 6. Conclusions

This study developed a new financial performance analysis model for football clubs. Using data from the period 2016–2018, and applying two different neural network methods in the creation of the financial performance analysis model to achieve a robust model, such as multilayer perceptron and the quantum neural network, this last methodology was the one that obtained the highest levels of precision. The most significant factors to explain financial performance were the variables of liquidity, leverage, and sporting performance. Some of them were the most common variables in our results. However, the reputation variable also showed some significance, which appeared on several occasions.

On the other hand, it was possible to increase the predictive capacity shown by the previous literature after the use of different neural network methodologies, after obtaining precisions from a precision range of 90.57–94.53%. Regarding the determining factors of financial performance, it detected new significant variables to consider in financial performance models for football clubs, allowing a high level of stability in the models developed over forecasting horizons of *t* + 1 and *t* + 2. In contrast to previous research, this study was able to expand the analysis of financial performance in football clubs beyond the accuracy and error results. The results identified a set of significant variables for each methodology applied and for each standard dependent variable, but some of these variables were recurrent in most models. This made an essential contribution to the field of corporate finance in football clubs. The conclusions were relevant to central executives, investors, managers, and other stakeholders in the football industry, who were generally interested in knowing which indicators provide reliable, accurate, and potential forecasts of performance evolution. Our study suggests new explanatory significant variables to allow these agents to analyze financial performance phenomena in football clubs. This research also provided a new financial performance analysis model developed for the football industry using two neural networks methods, being the QNN the most accurate, thus contributing to existing knowledge in the field of empirical corporate finance, and especially, neural networks. This new model can be used as a reference to improve decision-making in football club management.

In summary, this study provides a significant opportunity to contribute to the field of corporate finance’ football clubs, since the results obtained had significant implications for the future decisions of football club managers, making it possible to avoid big change events of the trend of financial performance and the potential associated risks. It also helps these agents send warning signals to football clubs and avoid massive losses derived from a decrease in the performance of the club. Further research in this field includes the financial performance models other new variables as macroeconomic and regulatory considering credit conditions and financial fair play UEFA’s regulations.

## Figures and Tables

**Table 1:** Differences and advantages of this study regarding previous works.

Differences	Advantages
This study uses a data sample of more than 200 European football clubs, while most of the previous works are focused on a country club’s analysis. Additionally, it uses two perspectives of financial performance such as return on net worth and return on capital employed, to verify the profitability achieved with the club’s funds as well as the profitability of the club’s activity. Ratios of a different nature are used, such as sports, corporate governance, financial, and reputation, expanding the types of ratios normally used in previous literature. Two neural network methodologies are applied to model and analyze the financial performance of any club deeply and individually, while most of the previous literature applies multicriteria methodology where they perform financial performance rankings, our study creates a new model for analyzing the clubs individually.	This study uses a larger sample geographically, which makes it possible to obtain a greater experience on the financial performance of football clubs. This study uses two neural network methodologies, which have had great results in finance. One of them is the popular multilayer perceptron, easy to implement, and the second variant is the quantum neural network, a novel technique that achieves great precision results. Thanks to this type of methodology and the construction of the sample, each club can be analyzed individually and thoroughly from our model. It is possible to identify financial performance weaknesses thanks to the inclusion in the model of different types of exploratory ratios.

**Table 2:** Independent variables.

Attribute	Code	Variables	Expected Sign
Corporate Governance Factors	I1	Institutional Ownership (binary)	−
	I2	Nº of Shareholders	+
	I3	Nº of Members of the Board of Directors	+
	I4	CEO Duality (binary)	−
Corporate Reputation Factors	R1	Facebook (number of followers)	+
	R2	Instagram (number of followers)	+
	R3	Twitter (number of followers)	+
Sporting Performance Factors	P1	Main Club of the City (binary)	+
	P2	Population of the City	+
	P3	Average Attendance at the Stadium	+
	P4	Accumulated points	+
	P5	Promotion/Relegation	+
	P6	Division	−
	P7	Performance Ratio (Szymanski Ranking ^1^)	+
	P8	Wage Bill (in EUR million)	+
Financial Factors	F1	Current Liabilities/Current Assets	−
	F2	Total Debt/Total Assets	−
	F3	Total Debt/Total Revenue	−
	F4	Expenses on Players/Operating Revenue	−
	F5	Working Capital/Total Assets	+
	F6	Retained Earnings/Total Assets	+
	F7	EBIT/Total Assets	+
	F8	Sales/Total Assets	+
	F9	Total Liabilities/Total Assets	−
	F10	Total Liabilities/Equity	−
	F11	Short-term Liabilities/Equity	−
	F12	Fixed Assets/Equity	+/−
	F13	Net Profit/Number of Shares	+
	F14	Net Capital/Equity	+
	F15	EBIT/Sales	+
	F16	Net Income Growth	+
	F17	Net Sale Growth	+
	F18	Asset Growth	+/−
	F19	Liabilities Growth	−
	F20	Debt Coverage Ratio	+

^1^ Szymanski Ranking = −ln(*p*/43 − *p*). The total of clubs that participate in the first and second division is 42, adding one more counting to the club with which you are working. The term “*p*” defines the final position the club achieved at the end of the season.

**Table 3:** Descriptive statistics.

	Mean	SD
**RONW**	0.104	0.092
**ROCE**	0.023	0.037
**I1**	0.417	0.489
**I2**	6.509	9.18
**I3**	18.516	11.683
**I4**	0.5	0.575
**R1**	3,114,528	82,693.391
**R2**	1,835,685	57,959.198
**R3**	974,404	26,942.458
**P1**	0.575	0.494
**P2**	927,451.696	2378.519
**P3**	21,393.802	158.461
**P4**	54.865	15.841
**P5**	−0.004	0.435
**P6**	1.499	0.674
**P7**	1.514	0.961
**P8**	183.567	15.019
**F1**	2.989	4.004
**F2**	0.683	0.395
**F3**	2.186	1.721
**F4**	1.206	1.38
**F5**	0.499	0.626
**F6**	−0.093	0.515
**F7**	−0.024	0.259
**F8**	0.735	0.924
**F9**	0.943	0.907
**F10**	15.808	10.854
**F11**	13.372	7.411
**F12**	4.457	5.328
**F13**	0.787	2.077
**F14**	13.267	12.291
**F15**	−0.012	0.774
**F16**	−0.015	0.567
**F17**	0.437	1.104
**F18**	0.116	0.793
**F19**	0.109	0.893
**F20**	0.151	0.567

**Table 4:** Results of accuracy evaluation: return on net worth (RONW).

Sample	MLP			QNN		
	Accuracy (%)	RMSE	MAPE	Accuracy (%)	RMSE	MAPE
Training	92.11	0.68	0.31	95.38	0.49	0.22
Validation	91.34	0.74	0.38	94.41	0.55	0.28
Testing	90.57	0.79	0.42	93.53	0.58	0.34

**Table 5:** Results of accuracy evaluation: return on capital employed (ROCE).

Sample	MLP			QNN		
	Accuracy (%)	RMSE	MAPE	Accuracy (%)	RMSE	MAPE
Training	93.27	0.61	0.27	96.17	0.42	0.18
Validation	92.58	0.67	0.32	95.41	0.46	0.24
Testing	91.46	0.75	0.39	94.53	0.51	0.30

**Table 6:** Results of accuracy evaluation: significant variables and normalized impact.

	MLP		QNN	
Dependent Variable	Significant Variables	Normalized Impact (%)	Significant Variables	Normalized Impact (%)
**RONW**	I1	54	I4	42
	R1	39	R1	51
	P1	76	P2	64
	P8	47	P8	34
	F1	42	F1	38
	F2	64	F2	72
	F4	52	F5	63
	F14	58	F14	44
**ROCE**	I4	62	I4	51
	R1	47	R2	60
	P7	82	P7	73
	P8	38	P8	42
	F1	34	F1	35
	F3	73	F3	63
	F7	56	F7	47
	F14	63	F12	57
	F20	48	F20	36

**Table 7:** Multiple-step ahead forecasts in forecast horizon = t+1 and t + 2.

	**RONW**					
**Horizon**	**MLP**			**QNN**		
	**Accuracy (%)**	**RMSE**	**MAPE**	**Accuracy (%)**	**RMSE**	**MAPE**
*t* + 1	88.32	0.95	0.56	91.85	0.87	0.59
*t* + 2	86.16	1.14	0.78	89.41	1.03	0.67
	**ROCE**					
**Horizon**	**MLP**			**QNN**		
	**Accuracy (%)**	**RMSE**	**MAPE**	**Accuracy (%)**	**RMSE**	**MAPE**
*t* + 1	89.67	0.84	0.49	92.38	0.80	0.52
*t* + 2	87.21	0.91	0.72	89.41	0.92	0.63

## Appendix A

**Table A1:** Clubs by National League.

National League	Number
Belgium	12
Bulgaria	3
Croatia	2
Cyprus	1
Czech Republic	2
Denmark	7
England	41
France	22
Germany	29
Greece	5
Italy	27
Netherlands	12
Norway	3
Poland	5
Portugal	7
Romania	2
Russia	1
Scotland	6
Spain	37
Sweden	2
Switzerland	1
Turkey	2
Ukraine	4

## References

[1] Keskin A.İ., Dincer B., Dincer C. (2020). Exploring the Impact of Sustainability on Corporate Financial Performance Using Discriminant Analysis. Sustainability.

[2] Union of European Football Associations, UEFA (2017). Financial Report 2016/2017.

[3] Rohde M., Breuer C. (2016). Europe’s Elite Football: Financial Growth, Sporting Success, Transfer Investment, and Private Majority Investors. Int. J. Financ. Stud..

[4] Andreff W. (2018). Financial and Sporting Performance in French Football Ligue 1: Influence on the Players’ Market. Int. J. Financ. Stud..

[5] Deloitte (2018). Annual Review of Football Finance.

[6] Union of European Football Associations, UEFA (2018). Club Licensing and Financial Fair Play Regulations. https://documents.uefa.com/viewer/document/MFxeqLNKelkYyh5JSafuhg.

[7] Union of European Football Associations, UEFA (2009). Club Licensing and Financial Fair Play Regulations. https://www.uefa.com/MultimediaFiles/Download/Tech/uefaorg/General/01/58/53/59/1585359_DOWNLOAD.pdf.

[8] Galariotis E., Germain C., Zopounidis C. (2018). A combined methodology for the concurrent evaluation of the business, financial and sports performance of football clubs: The case of France. Ann. Oper. Res..

[9] Lopez-Busto A., García-Unanue J., Gomez-Gonzalez C., Barajas Alonso Á., Gallardo L. (2016). Incidencia De Los Resultados Deportivos, Las Variables Económicas Y Administrativas En El Rendimiento Financiero De Los Clubes De Fútbol (Sports Scores, Financial and Administrative Variables on the Financial Performance of Football Clubs). CCD Cult. Cienc. Deporte.

[10] Giovanni P. (2017). The Football Team Composition Problem: A Stochastic Programming approach. J. Quant. Anal. Sports.

[11] Iconomescu T.M. Top Romanian Football Clubs Economic and Sport Performance Analysis. Proceedings of the 33rd International Business Information Management Association Conference.

[12] Da Costa Jahara R., Mello J.A.V.B., da Gama Afonso H.C.A. (2016). Proposal for Standard Index and Analysis of Financial Performance in 2014 of Brazilian Soccer Clubs of Serie A. Podium Sport Leis. Tour. Rev..

[13] Andreff W. (2014). French Professional Football: How Much Different?. Handbook on the Economics of Professional Football.

[14] Carmichael F., McHale I., Thomas D. (2010). Maintaining Market Position: Team Performance, Revenue and Wage Expenditure in the English Premier League. Bull. Econ. Res..

[15] Dimitropoulos P.E., Limperopoulos V. (2004). Player contracts, athletic and financial performance of the Greek football clubs. Glob. Bus. Econ. Rev..

[16] He H., Zhao J., Sun G. (2019). Prediction of MoRFs in Protein Sequences with MLPs Based on Sequence Properties and Evolution Information. Entropy.

[17] Singh K.J., Thongam K., De T. (2016). Entropy-Based Application Layer DDoS Attack Detection Using Artificial Neural Networks. Entropy.

[18] Yeung D.S., Cloete I., Shi D., Ng W.W.Y. (2010). Sensitivity Analysis for Neural Networks.

[19] Alaminos D., Esteban I., Salas M.B., Callejón A.M. (2020). Quantum Neural Networks for Forecasting Inflation Dynamics. J. Sci. Ind. Res..

[20] Bae J.K. (2012). Predicting financial distress of the South Korean manufacturing industries. Expert Syst. Appl..

[21] Alaminos D., Fernández M.A. (2019). Why do football clubs fail financially? A financial distress prediction model for European professional football industry. PLoS ONE.

[22] Lamothe-Fernández P., Alaminos D., Lamothe-López P., Fernández-Gámez M.A. (2020). Deep Learning Methods for Modeling Bitcoin Price. Mathematics.

